# Pitfalls of PCR-RFLP in Detecting SARS-CoV-2 D614G Mutation

**DOI:** 10.1055/s-0041-1735556

**Published:** 2022-06-13

**Authors:** Kok-Siong Poon, Karen Mei-Ling Tan

**Affiliations:** 1 Department of Laboratory Medicine, National University Hospital, Singapore, Singapore

Recent work by Hashemi et al reported the development of a polymerase chain reaction-restriction fragment length polymorphism (PCR-RFLP) assay in lieu of the standard sequencing-based assay for detection of D614G mutation in the spike gene of severe acute respiratory syndrome coronavirus 2 (SARS-CoV-2). 1 However, an error in numbering codon and targeting nucleotide change at the wrong position resulting in misidentification of “V615V” as “D614G” was picked up only after publication. 2 In this letter, we discussed several design issues which are crucial when developing the PCR-RFLP assay targeting the D614G mutation.

**Fig. 1:**
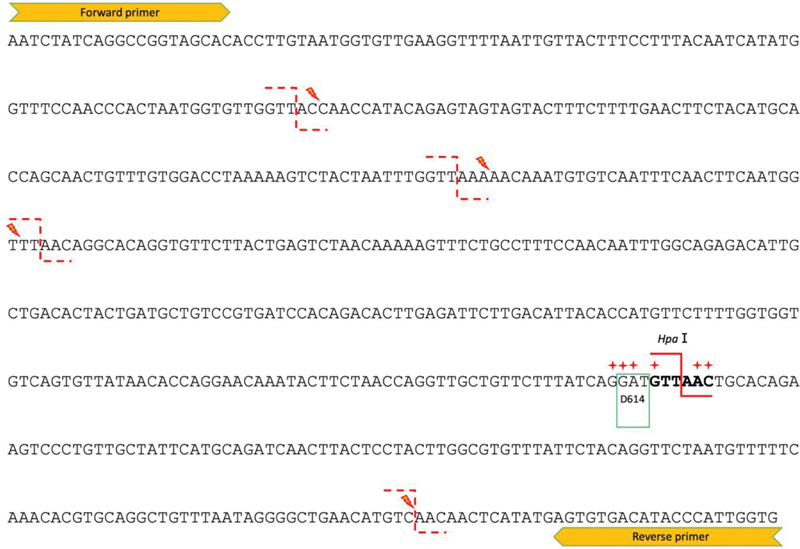
Nucleotide sequence of the 590bp polymerase chain reaction product amplified by the forward and reverse primers in the study by Hashemi et al. Codon 614 is boxed. Solid red line shows the *Hpa* I restriction site (GTTAAC). Red crosses indicate the known variants in close proximity to D614 codon reported in University of California Santa Cruz database which could abolish the cut site targeted by the *Hpa* I restriction enzyme. Lightning sign indicates the four potential *Hpa* I restriction site (dash line) after single nucleotide change. Bold sequences correspond to g.23405 to g.23410 of NC_045512.2.

PCR-RFLP has been a versatile molecular tool in molecular biology research and clinical diagnostics since its invention more than three decades ago. 3 Taking advantages from PCR for primer-dependent sensitivity and specificity in producing abundant amplicons from the nucleic acid targets, the downstream RFLP generates unique digestion profiles by using an appropriate restriction enzyme. Upon reverse transcription into cDNA, the primer designed by Hashemi et al amplifies a 590bp PCR product from the SARS-CoV-2 genomic region encoding the spike protein, in which codon 614 is located ( Fig. 1 ). In their article, Hashemi et al referenced the genomic sequence of isolate SARS-CoV-2/human/USA/WA-UW61/2020 - MT252819.1. In fact, NC_045512.2 should have been used since it is the standard reference sequence (RefSeq) for SARS-CoV-2. 4 The nucleotide position, 1845 targeted by Hashemi et al could have been easily verified by dividing it by three (codon triplet), and that is translated to codon 615 instead of 614. In D614G, the amino acid change of aspartic acid to glycine is mediated by an A > G transition resulting in GAT > GGT. In the article, it was mentioned as T to G, although this seems to be valid only with the wrong assumption of GAT > GGG. Hence, verification of the codon is important when identifying the target sequence to design a PCR-RFLP assay in this technical context. 5

We identified some potential pitfalls after further scrutinizing the assay design by Hashemi et al. The restriction enzyme *Hpa* I with the specific recognition sequence GTTAAC was chosen to cleave the PCR product ( Fig. 1 ). Mining of nucleotide variants from contemporary sequence submissions 6 7 returned at least three mutations reported within the genomic region targeted by *Hpa* I in this PCR amplicon. These mutations abolish the restriction site g.23405 to g.23410 and render the PCR product undigestable by this 6bp-cutter ( Fig. 1 ). Hence, specificity of this assay is further challenged by other mutations even if the authors had meant to target the nucleotide change resulting in V615V. Likewise in the influenza A virus, a synonymous change adjacent to the oseltamivir resistance mutation targeted by many PCR-based assays including PCR-RFLP was previously reported to interfere the assay's design and performance. 8 Hence checking the database for common reported variants present in the restriction site sequence is important when designing a PCR-RFLP assay.

The SARS-CoV-2 is an RNA virus with high mutability. 9 10 Within the 590bp amplified region, there are four vulnerable sites which require only single mutation to transform them into a *Hpa* I restriction site ( Fig. 1 ). The current sequence data in the databases 6 7 have not revealed a mutational event in any of the above-mentioned sites. However, emerging mutations may potentially complicate result interpretation of this assay. The strategy with PCR-RFLP is risky since the nucleotides adjacent to D614 are seemingly mutational hotspots ( Fig. 1 ). The utilization of a potentially faulty assay would have negative impact on the epidemiological study of SARS-CoV-2 if the error had been unnoticed. We hence call for vigilance in assay design for other nucleotide variants of interest in light of this ongoing SARS-CoV-2 pandemic.
